# Tris(2,4,6-trimethoxyphenyl)phosphine (TTMPP): Efficient Catalysts for the Cyanosilylation and Cyanocarbonation of Aldehydes and Ketones

**DOI:** 10.3390/molecules14093353

**Published:** 2009-09-02

**Authors:** Satoru Matsukawa, Izumi Sekine, Ayumi Iitsuka

**Affiliations:** Department of Science Education, Faculty of Education, Ibaraki University, Ibaraki, 310-8512 Japan

**Keywords:** organocatalysis, cyanosilylaton, cyanohydrin carbonate, phosphines, cyanation

## Abstract

A variety of aldehydes and ketones were transformed to their corresponding cyanohydrin silyl ethers in good to excellent yields in the presence of 1-5 mol% of tris(2,4,6-trimethoxyphenyl)phosphine (TTMPP). Cyanohydrin carbonates were also readily prepared using 5-10 mol% of TTMPP as an organocatalyst.

## Introduction

The addition of trimethylsilyl cyanide (TMSCN) to carbonyl compounds is one of the most popular strategies to afford cyanohydrins, which can be conveniently converted into various important polyfunctionalized building blocks for the synthesis of many natural products and bioactive molecules, including α-hydroxyl carbonyl compounds and β-amino alcohols [1,2,3,4,5,6,7]. Consequently, many catalytic systems, such as metal salts, organocatalysts, inorganic solid acids and bases, have been developed to enhance the efficiency of this transformation. Organophosphorous compounds have also been employed. Mukaiyama reported that Bu_3_P and Ph_3_P act as catalysts for the cyanosilylation with aldehydes [8]. Plumet [9,10] and Tian [11] independently reported that phosphonium salts-catalyzed cyanosilylation with aldehydes and ketones. Verkade’s base (cycloazaphosphine) also acts as a good catalyst in this reaction [12]. In search of other useful organophosphorous catalysts, we have now examined the use of a highly basic phosphine, tris(2,4,6-trimethoxyphenyl)phosphine (TTMPP), as a catalyst.

TTMPP is known to be a highly basic phosphine owing to its extensive methoxy-substitution [13]. Based on this property, some unique catalytic reactions have been reported [14,15,16,17,18,19]. We have also reported that TTMPP acts as a good Lewis base catalyst in the reaction with silylated nucleophiles via O-Si and C-Si bond activation [20,21,22,23,24,25]. Herein we wish to report an efficient catalytic cyanosiyliation of various aldehydes and ketones using TTMPP as a catalyst.

## Results and Discussion

Initially, the reactions of different aldehydes with trimethylsilyl cyanide in the presence of 1 mol% of TTMPP in DMF at room temperature were examined. For both aromatics having an electron-donating or -withdrawing group and aliphatic aldehydes, the reaction proceeded quite smoothly and the desired products were obtained at high yield within 30 min (Table 1). The product was obtained in lower yield when other phosphines, such as Bu_3_P, Ph_3_P and TMPP were used instead of TTMPP (Table 1, entries 1 vs. 10-12). The reaction also smoothly proceeded in THF. However, the reactions performed in MeCN and toluene were inferior when compared to those in DMF and THF.

**Table 1:** Cyanosilylation of various aldehydes.^[a]^

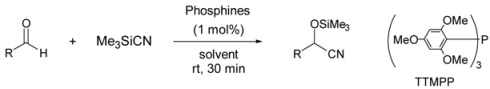				
Entry	Phosphines	Aldehyde	solvent	Yield (%)
1	TTMPP	C_6_H_5_CHO	DMF	98
2		4-CH_3_OC_6_H_4_CHO		99
3		4-ClC_6_H_4_CHO		95
4		4-NO_2_C_6_H_4_CHO		98
5		α-Naphthaldehyde		92
6		β-Naphthaldehyde		90
7		C_8_H_17_CHO		95
8		C_6_H_5_CH_2_CH_2_CHO		92
9		*cyclo*-C_6_H_11_CHO		93
10	TMPP^[b]^	C_6_H_5_CHO		45
11	Ph_3_P			30
12	*^n^*Bu_3_P			68
13	TTMPP		THF	95
14			CH_3_CN	55
15			toluene	30

^[a]^ Reactions were carried out on a 0.5 mmol scale with 1.2 equiv of TMSCN. ^[b]^ Tris(4-methoxyphenyl)phosphine.

**Table 2:** TTMPP-Catalyzed cyanosilylation of various ketones.^[a]^

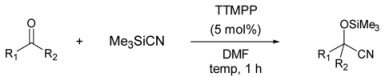			
Entry	Ketones (3)	Temp (°C)	Yield (%)
1	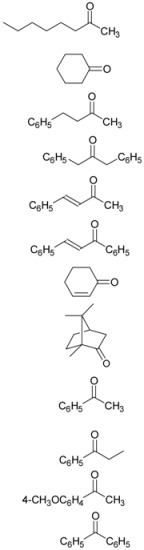	r.t.	97
2^[b], [c]^			99
3			95
4			93
5			98
6			90
7			93
8			94
9			76
10^[d]^		r.t.	trace
11		50 °C	97
12			96
13			95

^[a]^ Reactions were carried out on a 0.5 mmol scale with 1.2 equiv of TMSCN. ^[b]^ 1 mol% of TTMPP was used. ^[c]^ Reaction time: 5 h. ^[d]^ Reaction time: 24 h.

Next, we investigated the scope of this TTMPP-catalyzed reaction of ketones (Table 2). In the case of aliphatic ketones, the reaction proceeded smoothly in the presence of 5 mol% of TTMPP in DMF at room temperature. Good results were obtained for both acyclic and cyclic ketones. This reaction also proceeded smoothly when 1 mol% of TTMPP was used. Only 1,2-addition products were observed for unsaturated ketones. On the other hand, for the typical aromatic ketone acetophenone, only a trace amount of the product was obtained at room temperature even after 24 h. Then, the reaction was carried out at the elevated temperature. The desired product was obtained in 95% yield at 50 °C in DMF within 1 h. Under the same conditions, a variety of aromatic ketones were transformed to their corresponding cyanohydrin silyl ethers in high yield.

Cyanocarbonation [26,27,28,29,30,31,32,33] of aldehydes and ketones with cyanoformate was also examined (Table 3). Cyanohydrin carbonates are configurationally stable and significantly less prone to hydrolysis than cyanohydrin trimethylsilyl ethers. We examined the scope of this TTMPP-catalyzed reaction of aldehydes and ketones using methyl cyanoformate. Good results were obtained for both aromatics having an electron-donating or -withdrawing group and aliphatic aldehydes in the presence of 5 mol% of TTMPP in THF at room temperature. The reaction also proceeded smoothly with various aliphatic ketones in the presence of 10 mol% TTMPP, but was unsuccessful with aromatic ketones under these conditions.

**Table 3:** TTMPP-Catalyzed cyanocarbonation of aldehydes and ketone.^[a]^

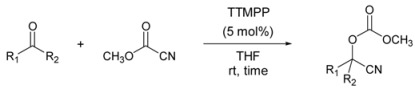			
Entry	Carbonyl Compound (3)	Time (h)	Yield (%)
	C_6_H_5_CHO	1 h	98
2	4-CH_3_OC_6_H_4_CHO	4 h	85
3	4-ClC_6_H_4_CHO		95
4	4-BrC_6_H_4_CHO		98
5	4-NO_2_C_6_H_4_CHO		88
6	α-Naphthaldehyde	1 h	98
7	β-Naphthaldehyde		85
8	C_8_H_17_CHO	4 h	87
9	C_6_H_5_CH_2_CH_2_CHO		92
10	*cyclo*-C_6_H_11_CHO		88
11^[b]^	C_6_H_13_COCH_3_	20 h	75
12^[b]^	C_6_H_5_CH_2_CH_2_COCH_3_		85
13^[b]^	C_6_H_5_COCH_3_	24 h	5
14^[b], [c]^		48 h	10

^ [a]^ Reactions were carried out on a 0.5 mmol scale with 1.5 equiv of methyl cyanoformate; ^[b]^ 10 mol% of TTMPP was used; ^[c]^ At 50 °C.

## Experimental

### General

All reactions were performed under an argon atmosphere using oven-dried glassware. Flash column chromatography was performed using silica gel Wakogel C-200. Preparative thin-layer chromatography was carried out on silica gel Wakogel B-5F. Dehydrate DMF, THF, toluene and CH_3_CN were purchased from Wako Chemical. Other commercially available reagents were used as received without further purification. ^1^H NMR and ^13^C NMR spectra were recorded on a JEOL JMN-GS400 spectrometer (399.65 MHz for ^1^H and 100.40 MHz for ^13^C).

### General Procedure for the TTMPP-Catalyzed Cyanosilylation of Aldehydes and Ketones

To a solution of TTMPP (0.05 mmol) in DMF (1 mL) was added aldehyde or ketone (0.5 mmol) and trimethylsilyl cyanide (0.60 mmol) at room temperature. After stirring for 30 min, the resultant mixture was quenched with water. The mixture was extracted with EtOAc and organic layer was washed with brine and dried over anhydrous Na_2_SO_4_, and evaporated. The crude mixture was purified by column chromatography on silica gel (EtOAc-hexane = 1:10) to give the corresponding product. The products are all known compounds identified by their spectroscopic data and comparison with literature values [8,9,10,11,12,34]. The purity of the product was confirmed by ^1^H NMR analysis.

### General Procedure for the TTMPP-Catalyzed Cyanocarbonation of Aldehydes

To a solution of TTMPP (0.05 mmol) in THF (1 mL) was added aldehyde (0.5 mmol) and methyl cyanoformate (0.75 mmol) at room temperature. After stirring for 30 min, the resultant mixture was quenched with water. The mixture was extracted with Et_2_O and organic layer was washed with brine and dried over anhydrous Na_2_SO_4_, and evaporated. The crude mixture was purified by column chromatography on silica gel (EtOAc-hexane = 1:3) to give the corresponding product. The products are all known compounds identified by their spectroscopic data and comparison with literature values [27,28,29,30,31,32,33]. The purity of the product was confirmed by ^1^H NMR analysis.

*α-(Trimethylsilyloxy)phenylacetonitrile*: ^1^H-NMR (400 MHz, CDCl_3_) δ 0.21 (s, 9H), 5.45 (s, 1H), 7.35 – 7.50 (m, 5H); ^13^C NMR (100 MHz, CDCl_3_) δ −0.3, 63.4, 119.1, 126.0, 128.4, 129.0, 136.0.

*2-Trimethylsilyloxy-2-phenylpropanenitrile*: ^1^H-NMR (400 MHz, CDCl_3_) δ 0.17 (s, 9H), 1.85 (s, 3H), 7.35 – 7.56 (m, 5H); ^13^C NMR (100 MHz, CDCl_3_) δ 1.0, 33.5, 71.5, 121.3, 124.8, 128.4, 141.9.

*α-(Methoxycarboxy)phenylacetonitrile*: ^1^H-NMR (400 MHz, CDCl_3_) δ 3.87 (s, 3H), 6.28 (s, 1H), 7.39 – 7.55 (m, 5H); ^13^C NMR (100 MHz, CDCl_3_) δ 55.9, 66.6, 115.6, 127.8, 129.1, 130.6, 131.1, 153.9.

## Conclusions

In conclusion, we demonstrated that TTMPP catalyzes cyanosilylation reactions using TMSCN. TTMPP effectively activated the C-Si bond of TMSCN, and the reaction proceeded smoothly to afford the corresponding products. This reaction can be applied to wide variety of aldehydes and ketones. Cyanohydrin carbonates could also be readily prepared with methyl cyanoformate using 5-10 mol% of TTMPP as catalyst.
